# Perfectionism as Possible Predictor for Treatment Success: Preliminary Data From Metacognitive Training for Depression and Suicidal Ideation in an Inpatient Sample

**DOI:** 10.1002/jclp.70027

**Published:** 2025-08-07

**Authors:** Nathalie Claus, Jakob Scheunemann, Sönke Arlt, Judith Peth, Jürgen Gallinat, Barbara Cludius, Lena Jelinek

**Affiliations:** ^1^ Department of Psychology, LMU Munich Division of Clinical Psychology and Psychological Treatment Munich Germany; ^2^ Division of Clinical Psychology and Psychotherapy in Adults Institute of Psychology University of Bremen Bremen Germany; ^3^ Department of Psychiatry and Psychotherapy University Medical Center Hamburg‐Eppendorf Hamburg Germany

**Keywords:** depression, metacognitive training, perfectionism, psychopathology, suicidal ideation, treatment outcome

## Abstract

**Objectives:**

In the treatment of depression and suicidal ideation, perfectionism has emerged as a possible predictor of treatment outcome. Some data suggest that cognitive‐behavioral therapy outcomes are poorer for more perfectionistic patients. However, findings so far neglect the multidimensionality of perfectionism, and research has yet to be extended to newer treatment approaches.

**Methods:**

The current study comprised secondary analysis of data from inpatients in treatment for depression. We administered measures of perfectionistic concerns and perfectionistic strivings as well as depression and suicidal ideation severity. Patients received 4 weeks of metacognitive training for depression and suicidal ideation (D‐MCT/S) in a group setting, alongside a comprehensive inpatient treatment. Hierarchical data over time was submitted to multi‐level analysis.

**Results:**

Perfectionistic concerns at baseline negatively predicted depressive symptoms but not suicidal ideation across time points. In exploratory analysis including perfectionistic strivings as an additional predictor, perfectionism dimensions no longer predicted depressive symptoms. A reduction of perfectionistic concerns during treatment did not predict symptoms at follow‐up. However, sensitivity analyses revealed significant differences in results based on the choice of symptom control variables.

**Conclusions:**

These results suggest that initial perfectionistic concerns may not prevent patients with depression and suicidal ideation from benefitting from metacognitive treatment. However, the contrast to previous findings may also be explained by differing results when separating the effects of perfectionistic concerns and perfectionistic strivings, as well as the choice of symptom control measure, or limited statistical power. Limitations and avenues for future research are discussed.

## 1 Introduction

Perfectionism has been proposed as a predictor of treatment response in cognitive‐behavioral therapy (CBT) in various mental disorders (De Cuyper et al. 2019; Kyrios et al. 2015; Mitchell et al. 2013), including the treatment of depression (Blatt et al. 1995, 1998; Hawley et al. 2022; Jacobs et al. 2009; Zuroff et al. 2000). However, the majority of previous studies have not considered the multidimensionality of perfectionism, which combines perfectionistic strivings and perfectionistic concerns (Bieling et al. 2004; Stoeber and Otto 2006). Whereas perfectionistic strivings constitute striving for flawless results, perfectionistic concerns comprise excessive self‐criticism when those results are not achieved. Additionally, perfectionism is cross‐sectionally associated with suicidal ideation (Smith et al. 2018), yet a possible impact of perfectionism on the treatment of suicidal ideation remains understudied. Thus, the aim of this study was to examine the effect of perfectionistic concerns on the treatment of depressive symptoms and suicidal ideation, in an inpatient sample receiving a CBT‐based intervention (Metacognitive Training for depression and suicidal ideation, D‐MCT/S). The current study should be considered a preliminary investigation into the role of perfectionism in metacognitive treatments for depression.

Perfectionism has emerged as a possible predictor of CBT response. It has been shown to impede CBT outcomes across various disorders, such as anxiety disorders (Ashbaugh et al. 2007; Mitchell et al. 2013), eating disorders (Bizeul et al. 2001; De Cuyper et al. 2019; Sutandar‐Pinnock et al. 2003; Welch et al. 2020), and obsessive‐compulsive disorder (Chik et al. 2008; Kyrios et al. 2015; Manos et al. 2010). This detrimental impact on symptom reduction is also evident in depression. Baseline perfectionism negatively predicts reductions in depressive symptoms (Blatt et al. 1995, 1998; Hawley et al. 2022; Jacobs et al. 2009; Zuroff et al. 2000), with the detrimental effect on therapeutic outcome (i.e., clinician‐rated level of functioning) and treatment satisfaction persisting up to 18 months after the end of treatment (Blatt et al. 1998). Further, an improvement in perfectionism during treatment is associated with subsequent improvement in depressive symptoms (Hawley et al. 2006). These associations have been demonstrated for individual as well as group settings (Hawley et al. 2022; Hewitt et al. 2020). However, it is important to note that a majority of previous studies focusing on perfectionism in the treatment of depression relied on data from the same research program, the Treatment of Depression Collaborative Research Program (Blatt et al. 1995, 1998; Hawley et al. 2006; Marshall et al. 2008; Zuroff et al. 2000). Hence, generalizability of extant evidence may be limited, seeing as it is largely based on homogenous samples.

Nevertheless, one explanation for why perfectionism may hamper treatment lies in dysfunctional metacognitive beliefs about the benefits of perfectionism (e.g., “only by being strict with myself can I achieve my goals”) (Shafran et al. 2002). However, there is evidence that only some dimensions of perfectionism directly predict treatment outcome (Hewitt et al. 2020). This comprises one of several gaps in the literature which need to be addressed.

Firstly, aside from Hewitt et al. (2020), all remaining studies to date (Blatt et al. 1995, 1998; Hawley et al. 2006, 2022; Jacobs et al. 2009; Marshall et al. 2008; Zuroff et al. 2000) have used a subscale of the Dysfunctional Attitudes Scale (DAS) and measured perfectionism as a unidimensional construct. Whereas unidimensional perfectionism measures are not uncommon, such measures cannot differentiate between the two subdimensions commonly assumed to constitute perfectionism, namely perfectionistic strivings and perfectionistic concerns (Bieling et al. 2004; Stoeber and Otto 2006). Perfectionistic strivings encompass the setting of exceedingly high standards in striving for perfection (Gaudreau 2019), whereas perfectionistic concerns comprise excessive self‐criticism in the face of perceived failures to meet those standards (Bieling et al. 2004). The use of a measure which accounts for the multidimensionality of perfectionism would be prudent considering evidence that the two dimensions may differ in their associations with various psychopathology. Namely, correlations between the severity of symptoms, including depression, and perfectionistic concerns are markedly stronger than those with perfectionistic strivings (for meta‐analyses, see Limburg et al. 2017 and Callaghan et al. 2023 for cross‐sectional evidence, and see Smith et al. 2021 for longitudinal evidence). Perfectionistic concerns, as a dysfunctional scheme for self‐evaluation based on performance (Shafran et al. 2002), might reasonably feed into a depressive self‐view as a failure and corresponding feelings of shame and guilt (see Vulnerability Model of Perfectionism, e.g., Hewitt and Flett 2002). Perfectionistic strivings, on the other hand, must not necessarily impact an individual's self‐worth and corresponding mood, seeing as they do not encompass feelings of discrepancy and doubt (Stoeber and Gaudreau 2017; Stoeber and Otto 2006). Hence, perfectionistic strivings have yielded associations with both negative and positive outcomes before (Stoeber and Otto 2006). As a unidimensional measure, however, the perfectionism subscale of the DAS cannot differentiate between these two perfectionism dimensions. In fact, it indiscriminately includes items of both perfectionistic strivings (e.g., “a person should try to be the best at everything he/she undertakes”) and perfectionistic concerns (“I should be upset if I make a mistake”). Taken together, except for the study by Hewitt et al. (2020), no study has used a multidimensional scale to assess the effect on treatment outcome in patients with depression. The Frost Multidimensional Perfectionism Scale (FMPS; Frost et al. 1990) lends itself well to the question at hand. It assesses both perfectionistic strivings and perfectionistic concerns. The two dimensions can be measured using the FMPS subscales “personal standards” and “concern over mistakes”, respectively (Howell et al. 2020).

Secondly, perfectionism is closely related not only to depression, but to suicidal ideation as well. Both perfectionism dimensions show a strong link with suicidal ideation (for a meta‐analysis, see Smith et al. 2018). Additionally, perfectionistic concerns are associated with suicide attempts (Smith et al. 2018). Perfectionism has in fact been posited as one vulnerability factor for suicide (Flett et al. 2014; Roxborough et al. 2012). This seems particularly pertinent for the treatment of depression, since depression often goes along with suicidal ideation (Franklin et al. 2017). A third of individuals affected by depression are estimated to attempt suicide in their life (Dong et al. 2019). However, the role of perfectionism in the treatment of suicidal ideation is of yet vastly understudied. Beevers and Miller (2004) followed depressed inpatients after discharge and a period of outpatient treatment (including medication and CBT). Higher perfectionism measured during the inpatient stay was associated with higher suicidal ideation 6 months later. Similarly, in an adolescent sample receiving outpatient treatment (including medication and CBT), baseline perfectionism impeded improvement from suicidal ideation (Jacobs et al. 2009). Both studies used the DAS subscale to measure perfectionism, and treatments did not specifically target suicidal ideation. In addition, evidence from adolescent samples may not be fully transferrable to adult participants, seeing as adolescence has been discussed as a period of self‐consciousness which could be more prone to perfectionism (Flett et al. 2002).

Aside from “classic” CBT treatments, new treatments for depression have emerged in recent years, some of which aim to explicitly address suicidal ideation. One such treatment is Metacognitive Training for Depression (D‐MKT; Jelinek et al. 2023, with materials available at https://uke.de/depression), which is based on the MCT for psychosis (Moritz and Woodward 2007) and adapted for patients with depression. In general, MCT aims to develop more cognitive flexibility and thus reduce the stress caused by disorder‐specific cognitions. The original MCT for psychosis has shown small to moderate effects on symptoms (Meinhart et al. 2024; Penney et al. 2022) as well as targeted cognitions (Hotte‐Meunier et al. 2024; Melville et al. 2024) in meta‐analyses. The depression‐specific version of MCT, D‐MCT, is a standardized group treatment devised to alter depression‐specific cognitive biases (e.g., overgeneralization) and dysfunctional coping strategies (e.g., thought suppression). It also addresses perfectionism. In randomized controlled trials comparing D‐MCT with active controls as part of a comprehensive treatment program, D‐MCT has shown greater symptom reductions for patients with depression in both inpatient (Schneider et al. 2024), day patient (Jelinek et al. 2016, 2018), and outpatient settings (Moritz et al. 2025). The D‐MCT has recently been adapted to include two new modules on suicidal ideation (D‐MCT/S; Jelinek et al. 2021; Miegel et al. 2022). Based on the idea that certain metacognitions typical of highly perfectionistic individuals may be what hinders treatment success (Shafran et al. 2002), it is feasible that tackling these metacognitions would help such individuals benefit more from treatment. Whereas perfectionists are expected to severely criticize themselves after perceived failure (e.g., not completing a homework assignment “perfectly”), MCT explicitly frames mistakes as positive learning opportunities (Moritz and Woodward 2007). To date, it is unclear whether this approach may indeed limit the impeding effect of perfectionism on treatment.

The aim of the current study was to investigate perfectionism as a predictor of symptom outcome in MCT for depression and suicidal ideation (D‐MCT/S). We examined the effect of both baseline perfectionism and the change in perfectionism on treatment outcome. To this end, we used baseline, posttreatment, and follow‐up data from an existing dataset (Jelinek et al. 2021). D‐MCT/S was offered to inpatients with depression as a group intervention with eight sessions (4 weeks). Considering the structure of the data, we chose multi‐level analyses to allow for flexible analysis of changes over time and let individuals vary in their baseline and change scores (Curran et al. 2010). In extension of previous studies, we used the FMPS to assess perfectionistic concerns and perfectionistic strivings separately. In addition to depressive symptoms, we assessed suicidal ideation as a secondary treatment outcome.

Based on previous results from CBT research, we hypothesized that greater perfectionism at baseline would predict greater depressive symptom severity at posttreatment and 4‐week follow‐up (H1), controlling for symptom severity at baseline. We further expected that a greater reduction in perfectionism from baseline to posttreatment would predict lower depressive symptom severity at 4‐week follow‐up (H2), controlling for symptom severity at posttreatment. Both hypotheses were tested for depressive symptom severity (H1 and H2) and suicidal ideation (H3 and H4) as the respective outcomes. The study was preregistered before data analysis, at https://osf.io/2fzye.

## 2 Materials and Methods

### 2.1 Study Design

This study employed a repeated‐measurements design and used data from a previous uncontrolled treatment study described elsewhere (Jelinek et al. 2021; Miegel et al. 2022). Out of this data set, data from baseline, posttreatment (4 weeks after baseline), and 4‐week follow‐up (8 weeks after baseline) was used for those participants who had filled in perfectionism measures. Additionally, exploratory analysis included data from a separate project, an 18‐month prospective study which included 36 patients from this pre‐selected sample (Scheunemann et al. 2021). This data served as an 18‐month follow‐up for the current study.

### 2.2 Participants

A total of 49 patients with a primary diagnosis of depression were included for main analyses. Patients were recruited shortly after admission to the ward for affective disorders at the Clinic for Psychiatry and Psychotherapy of the University Center Hamburg‐Eppendorf. Enrollment took place between January 2016 and April 2017. All participants were assessed at baseline using the Diagnostic and Statistical Manual of Mental Disorders (DSM‐5; American Psychiatric Association 2013) to confirm diagnosis. To be included, a primary diagnosis of a depressive disorder was required, as well as age ranging from 18 to 65 years and sufficient German language skills. Additionally, participants with a diagnosis of schizophrenia, bipolar disorder, or mania, neurological disorders, or an IQ below 70 as estimated by a vocabulary test were excluded. Current analyses included only those patients who had been present for at least four group sessions (49 out of 59 assessed at baseline). Participants received 30€ as compensation for the first three time points (baseline, posttreatment, 4‐week follow‐up), with an additional 40€ for participation in the 18‐month follow‐up.

### 2.3 Intervention

All participants received a comprehensive inpatient treatment, including medication in most cases. In addition, they received D‐MCT/S group treatment consisting of eight sessions, with sessions lasting approximately 60 min each. The treatment was delivered bi‐weekly over the course of 4 weeks and used a slide‐based presentation. The group was conducted in an open‐group format, so that patients could join at any module.

Group treatment modules targeted dysfunctional cognitions and metacognitions considered relevant to depression. This included one module on self‐worth, in which sources of self‐worth and negative implications of perfectionism were discussed. Remaining modules addressed biased memory, black‐and‐white thinking, dysfunctional coping, jumping to conclusions, and emotions. This modified version of D‐MCT (D‐MCT/S) replaced two of the original modules with two new modules on suicidal ideation. These new modules aimed at modifying suicide‐specific dysfunctional cognitions and included topics such as helplessness and hopelessness, biases in decision‐making, and guilt, as well as the development of an emergency plan for suicidal crises. Details can be found elsewhere (Jelinek et al. 2021; Miegel et al. 2022; https://uke.de/depression).

### 2.4 Measures

#### 2.4.1 Frost Multidimensional Perfectionism Scale (FMPS)

The FMPS (Frost et al. 1990; German version: Stöber 1995) served as the predictor of interest. It consists of 35 items, all of which are rated on a 5‐point scale (1 = strong disagreement, to 5 = strong agreement), with its six subscales (concern over mistakes, doubts about actions, parental criticism, parental expectation, personal standards, order and organization) aiming to represent perfectionism as a multidimensional construct. The questionnaire is well established as a valid and reliable measure for perfectionism (Frost et al. 1990). Internal consistency in the current sample was excellent (Cronbach's α = 0.91).

For analyses, sum scores of two subscales were used. The 9‐item subscale “concern over mistakes” was used to assess perfectionistic concerns, with possible subscale scores ranging between 9 and 45 (Cronbach's α = 0.92). Items measure excessive mistake avoidance and an all‐or‐nothing attitude towards success/failure. The 7‐item subscale “personal standards” was used in exploratory analyses to assess perfectionistic strivings, with possible subscale scores ranging between 7 and 35 (Cronbach's α = 0.81). Items measure the setting of exceedingly high standards and the importance of being completely competent.

#### 2.4.2 Beck Depression Inventory‐II (BDI‐II)

Primary outcome was depression severity as measured by the BDI‐II (Beck et al. 1996; German version: Kühner et al. 2007). It is a well‐established self‐report measure of depressive symptom severity, with good psychometric properties (Kühner et al. 2007). Its 21 items, rated from 0 to 3, yield a score between 0 and 63. Internal consistency in the current sample was good (Cronbach's α = 0.89).

#### 2.4.3 Beck Scale for Suicide Ideation (BSS)

As a secondary outcome, the BSS (Beck and Steer 1991; German version: Kliem et al. 2017) was used. It is among the most widely used self‐report measures for suicidal thinking, with good psychometric properties (Kliem et al. 2017). The BSS consists of five screening items which determine whether an additional 16 items are to be answered or not, amounting to 21 items in total. Items are rated on a 3‐point scale (0–2). The final sum score is calculated using only the first 19 items, resulting in a score ranging between 0 and 38. Internal consistency in the current sample was good (Cronbach's α = 0.87).

#### 2.4.4 Hamilton Depression Rating Scale‐17 (HDRS‐17)

Preregistration included the 17‐item version of the HDRS (Hamilton 1960) as a control measure for depression severity. Due to conceptual concerns (e.g., Rogers et al. 2018), preregistered analyses were moved to the Supporting Information. The HDRS‐17 is considered the gold standard in clinician‐rated assessment of depression and has been shown to have good psychometric properties (Trajković et al. 2011). Scores range between 0 and 52. Internal consistency in the current sample was acceptable (Cronbach's α = 0.67). At 18‐month follow‐up, the HDRS‐17 was conducted via telephone.

### 2.5 Statistical Analyses

All statistical analyses were performed using R (R Core Team 2022), version 4.2.2.

#### 2.5.1 Data Exclusion and Missing Data

All available data was used. Imputation of missing values was performed using the R packages *naniar* (Tierney et al. 2021) and *zoo* (Zeileis and Grothendieck 2005), see Supporting Information for details.

#### 2.5.2 Multi‐Level Modeling

Due to the nested data structure, we used linear mixed models to test the predictive value of perfectionism for symptom severity. Each model had a two‐level structure, with repeated assessments modeled as level 1 and participants as level 2. Models were estimated using maximum‐likelihood estimation and included random subject‐level intercepts to account for nested observations. Starting from a basic model including only the intercept, complexity was added progressively in terms of fixed and random effects. Additionally, random slopes were added for each predictor to allow them to vary across participants. The error covariance matrix was modeled as autoregressive to account for repeated measures. At each step, a Likelihood Ratio Test with a level of significance of *α* = 0.05 was used to compare model‐fit and aid decisions about including specific terms. Thus, for each hypothesis, the model with the best fit was used to extract model parameters. Models were built using the R package *nlme* (Pinheiro et al. 2022). Assumptions of multi‐level modeling (linearity, homogeneity of variances, normal distribution of residuals) were checked by visual inspection (see Supporting Information for details).

First, to determine the level of nonindependence in the data (repeated measures nested in patients), we estimated the basic model for each hypothesis and calculated the intraclass correlation coefficient (ICC) at patient level. To test the effect of perfectionism on changes in depression after treatment (H1), we estimated a model with the depressive symptoms (BDI‐II total score) as the response variable and the following predictors: perfectionistic concerns (FMPS‐CM score, at baseline), depressive symptom severity (at baseline), time (weeks since baseline), and the interaction between perfectionistic concerns and time. We used the same to estimate the changes on the secondary outcome, namely, suicidal ideation (BSS) (H3). To investigate the effect of *change* in perfectionism on changes in depression after treatment (H2), we estimated a model with the depressive symptoms (BDI‐II total score) at follow‐up as the response variable, and change in perfectionistic concerns (FMPS‐CM score, from baseline to posttreatment) and depressive symptom severity (at posttreatment) as predictors. Again, we used the same to estimate the changes on the secondary outcome, suicidal ideation (BSS) (H4). Change in perfectionistic concerns was computed using residuals of a linear regression: PerfectionismPost_i_ ~ b_0_ + b_1_ * PerfectionismBaseline_i_. Assumed equations of multi‐level models can be found in the Supporting Information.

Please note: preregistration specified that when controlling for earlier symptom severity, different symptom scores be used than the outcome scores to circumvent merely calculating a measure's correlation with itself (e.g., when predicting symptom severity as measured by the BDI‐II, the HDRS‐17 would be used as the control measure). However, through the review process, conceptual concerns with this approach were raised (e.g., Rogers et al. 2018). Hence, the manuscript now presents sensitivity analyses using identical control measures as the main analyses. Meanwhile, the preregistered analyses were moved to the Supporting Information (see Table S2).

#### 2.5.3 Centering

The predictors perfectionistic concerns and symptom scores were grand‐mean centered, using the respective mean at baseline. Time was transformed to measure weeks since the baseline assessment (i.e., baseline = 0, posttreatment = 4, follow‐up = 8).

#### 2.5.4 Exploratory Analyses

To test whether perfectionistic strivings (FMPS‐PS) predicted treatment outcome beyond the predictive value of perfectionistic concerns (FMPS‐CM), we computed additional models for hypotheses 1 and 3 with perfectionistic strivings included as an additional predictor. Further, hypotheses 2 and 4, that is, the effect of change in perfectionistic concerns (FMPS‐CM) on depression or suicidal ideation after treatment, were tested using data from an additional follow‐up measurement at 18 months after treatment.

## 3 Results

### 3.1 Sample Description

Demographic and clinical characteristics of the sample are shown in Table 1 and Table 2, respectively. 65% of the sample attended at least seven out of eight group sessions. All participants received at least one of the two suicide‐related modules (with 69.4% attending both), and 85.7% of the sample received the module discussing self‐worth. In a data set including all participants assessed at baseline (*n* = 59), number of sessions attended was not significantly related to baseline perfectionism (FMPS‐CM: *p* = 0.11; FMPS‐PS: *p* = 0.43), baseline depression (BDI‐II: *p* = 0.57), suicidal ideation at baseline (BSS: *p* = 0.29), days since admission (*p* = 0.55), duration of illness (*p* = 0.52), education (*p* = 0.33), or age (*p* = 0.98).

**Table 1:** Demographic characteristics of sample at baseline.

	*M* (SD) or % Total sample (*N* = 49)
Age at enrollment in years^a^	41.8 (9.4)
Range	25‐62
Gender (female)^a^	57.1%
Education in years^b^^,^ c	16.6 (3.9)
Current psychopharmacological medication^a^	
Anti‐depressives^d^	6.1%
Anti‐psychotics^d^	4.1%
Combination^e^	81.6%
None	6.1%
Number of D‐MCT/S sessions attended^a^	6.9 (1.3)
Mean duration of illness in years^a^	11.9 (11.8)
Number of comorbidities^a^	
None	34.7%
One	36.7%
Two or more	28.6%

^a^ *n* = 49.

^b^ *n* = 48.

^c^ Total amount, including school, vocational training, university.

^d^ Monotherapy.

^e^ Combined medication of anti‐depressives and anti‐psychotics.

**Table 2:** Clinical characteristics of sample across time points.

	Baseline *M* (SD) or %	Posttreatment *M* (SD) or %	Follow‐Up (4 weeks) *M* (SD) or %	Follow‐Up (18 months) *M* (SD) or %
Clinician‐rated depressive symptoms (HDRS‐17)	20.5 (6.6)^a^	16.2 (6.5)^b^	14.8 (7.9)^d^	11.1 (8.8)^e^
Self‐rated depressive symptoms (BDI‐II)	34.2 (12.25)^a^	26.6 (13.5)^b^	26.9 (14.3)^c^	24.1 (16.2)^e^
Self‐rated suicidal ideation (BSS)	11.1 (10.8)^a^	8.8 (10.7)^b^	9.2 (10.7)^d^	8.5 (10.7)^e^
Self‐rated perfectionism (FMPS)	88.5 (24.1)^a^	90.9 (23.6)^f^	/	/
Perfectionistic Concerns (FMPS‐CM)	27.0 (9.1)^a^	28.2 (8.9)^f^	/	/
Perfectionistic Strivings (FMPS‐PS)	23.6 (6.1)^a^	24.6 (6.2)^f^	/	/

Abbreviations: HDRS‐17, Hamilton Depression Rating Scale; BDI‐II, Beck Depression Inventory‐II; BSS, Beck Suicide Ideation Scale; FMPS, Frost Multidimensional Perfectionism Scale; FMPS‐CM, Frost Multidimensional Perfectionism Scale, subscale “concern over mistakes”; FMPS‐PS, Frost Multidimensional Perfectionism Scale, subscale “personal standards”.

^a^ *n* = 49.

^b^ *n* = 47.

^c^ *n* = 46.

^d^ *n* = 45.

^e^ *n* = 36.

^f^ *n* = 29.

On average, participants at baseline assessment showed moderate depressive symptoms according to a clinical interview (HDRS‐17) and severe depressive symptoms according to a self‐report measure (BDI‐II). 30.6% of the sample endorsed no suicidal ideation (BSS) at baseline. Perfectionism scores (FMPS) were comparable to community samples (Egan et al. 2016), with slightly elevated scores for perfectionistic concerns (FMPS‐CM).

Paired *t*‐tests revealed significant reductions in depression (BDI‐II: *p* < 0.001, *d* = 0.88) and suicidal ideation (BSS: *p* < 0.01, *d* = 0.37) from baseline to posttreatment, whereas reductions in perfectionism were not significant over time (FMPS‐CM: *p* = 0.77; FMPS‐PS: *p* = 0.74).

### 3.2 Multi‐Level Modeling

Results of final models are presented in Table 3, with alpha adjusted to account for multiple comparisons (two separate models per time point; α = 0.05/2 = 0.025). Bivariate correlations between all variables are documented in the Supporting Information (see Table S1). The Supporting Information also holds a description of model‐building steps, including statistical values, and equations of the final models after step‐wise inclusion of predictors, interaction terms, and random slopes.

**Table 3:** Results of the final multi‐level models for main analyses.

	*n*	β^a^	*95% CI*	*SE*	*t*	*p*
H1: Dependent variable: depressive symptom severity (BDI‐II) across time	49					
Intercept		32.27	31.36–33.17	0.46	69.55	**< 0.001**
Perfectionistic concerns at baseline (FMPS‐CM)		−0.14	−0.25 to −0.03	0.06	−2.44	**0.019**
Depressive symptoms at baseline (BDI‐II)		1.01	0.93–1.10	0.04	24.22	**< 0.001**
Time		−1.05	−1.49 to −0.61	0.23	−4.65	**< 0.001**
H2: Dependent variable: depressive symptom severity (BDI‐II) at 4‐week follow‐up	29					
Intercept		33.55	29.95–37.15	1.85	18.14	**< 0.001**
Change in perfectionistic concerns (FMPS‐CM)		0.70	0.03–1.36	0.34	2.05	0.051
Depressive symptoms at post (BDI‐II)		0.77	0.49–1.05	0.14	5.33	**< 0.001**
H3: Dependent variable: severity of suicidal ideation (BSS) across time	49					
Intercept		10.71	9.25–12.16	0.74	14.44	**< 0.001**
Perfectionistic concerns at baseline (FMPS‐CM)		−0.04	−0.16 to 0.08	0.06	−0.70	0.488
Suicidal ideation at baseline (BSS)		0.87	0.77–0.97	0.05	17.43	**< 0.001**
Time		−0.32	−0.58 to −0.05	0.13	−2.35	**0.021**
H4: Dependent variable: severity of suicidal ideation (BSS) at 4‐week follow‐up	28					
Intercept		11.98	9.51–14.44	1.27	9.46	**< 0.001**
Change in perfectionistic concerns (FMPS‐CM)		0.26	−0.17 to 0.70	0.22	1.19	0.246
Suicidal ideation at post (BSS)		0.81	0.59–1.04	0.12	7.07	**< 0.001**

Abbreviations: FMPS_CM, Frost Multidimensional Perfectionism Scale, “concern over mistakes” subscale; FMPS_PS, Frost Multidimensional Perfectionism Scale, “personal standards” subscale; BSS, Beck Suicide Ideation Scale; BDI‐II, Beck Depression Inventory II.

^a^ β (= fixed effect) denotes magnitude of change in the outcome variable as the predictor increases by one point relative to grand‐mean at baseline. Bold *p* values denote significance below α = 0.025 (Bonferroni‐corrected for multiple comparisons).

#### 3.2.1 Effect of Baseline Perfectionism (FMPS‐CM) on Depressive Symptom Severity (Hypothesis 1)

The final model showed that baseline perfectionistic concerns significantly predicted depressive symptoms (BDI‐II) across time points; that is, greater perfectionistic concerns (FMPS‐CM) at baseline were associated with lower depressive symptoms (BDI‐II) across time points. Depressive symptoms (BDI‐II) at baseline and time since baseline significantly also predicted depressive symptoms (BDI‐II); that is, higher depressive symptoms at baseline (BDI‐II) were associated with higher depressive symptoms (BDI‐II) across time points, and with every week since baseline, depressive symptoms (BDI‐II) decreased. The interaction term was excluded before formal testing due to model fit concerns (see Supporting Information). Fixed effects explained 66% of variance, with the entire model (including random effects) explaining 91.4% (σ^2^ = 19.13, τ_00_ = 0.60, τ_11_ = 1.87, ρ_01_ = 0.97). This model used data from all 49 participants, with ICC = 0.75.

#### 3.2.2 Effect of Change in Perfectionism (FMPS‐CM) on Depressive Symptom Severity (Hypothesis 2)

The final model showed that pre‐post change in perfectionistic concerns (FMPS‐CM) did not significantly predict depressive symptoms (BDI‐II) at 4‐week follow‐up. Only depressive symptom severity (BDI‐II) at posttreatment significantly predicted depressive symptoms; that is, higher depression scores (BDI‐II) at posttreatment predicted higher depression scores (BDI‐II) at 4‐week follow‐up. Fixed effects explained 94.3% of variance (σ^2^ = 9.83, τ_00_ = 69.91, ICC = 0.88). This model used data from 29 participants (complete FMPS data at baseline and posttreatment as well as BDI‐II data at 4‐week follow‐up).

#### 3.2.3 Effect of Baseline Perfectionism (FMPS‐CM) on Suicidal Ideation (Hypothesis 3)

The final model showed that baseline perfectionistic concerns (FMPS‐CM) did not significantly predict suicidal ideation (BSS). However, both suicidal ideation (BSS) at baseline and time significantly predicted suicidal ideation (BSS); that is, more severe suicidal ideation (BSS) at baseline was associated with more severe suicidal ideation (BSS) across time points, and with every week since baseline suicidal ideation (BSS) decreased. The interaction term was excluded before formal testing due to model fit concerns (see Supporting Information). Fixed effects explained 76% of variance (σ^2^ = 28.20, τ_00_ = 62.84, ICC = 0.00). This model used data from all 49 participants. Since visual inspection revealed violated assumptions of variance homogeneity and normal distribution of residuals, a multi‐level model may not have been the ideal fit for the data.

#### 3.2.4 Effect of Change in Perfectionism (FMPS‐CM) on Suicidal Ideation (Hypothesis 4)

The final model showed pre‐post change in perfectionistic concerns (FMPS‐CM) did not significantly predict suicidal ideation (BSS) at 4‐week follow‐up. Only suicidal ideation (BSS) at posttreatment significantly predicted suicidal ideation (BSS) at 4‐week follow‐up; that is, more severe suicidal ideation (BSS) at posttreatment was associated with higher suicidal ideation (BSS) at 4‐week follow‐up. Fixed effects explained 94.8% of variance (σ^2^ = 4.93, τ_00_ = 35.06, ICC = 0.88). This model used data from 28 participants (complete FMPS data at baseline and posttreatment as well as BSS data at 4‐week follow‐up). Since visual inspection revealed violated assumptions of variance homogeneity and normal distribution of residuals, a multi‐level model may not have been the ideal fit for the data.

### 3.3 Exploratory Analyses

Preregistered analyses, using symptom control measures different to the outcome measures, were moved to the Supporting Information (see above, also Table S2). In those preregistered analyses, baseline perfectionistic concerns did not significantly predict either depressive symptoms (BDI‐II) or suicidal ideation (BSS) across time points. Pre‐post change in perfectionistic concerns (FMPS‐CM), however, did significantly predict depressive symptoms (BDI‐II), but not suicidal ideation (BSS) at 4‐week follow‐up; that is, with every point decrease in perfectionistic concerns (FMPS‐CM) from baseline to posttreatment, depressive symptoms (BDI‐II) at 4‐week follow‐up decreased.

Exploratory analyses as described above are also presented in the Supporting Information (Tables S3 and S4). Since these analyses are of an exploratory nature, alpha was not corrected for multiple testing. Results revealed that, when both perfectionism dimensions (FMPS‐CM, FMPS‐PS) were added as predictors within the same models, neither perfectionism dimension significantly predicted depressive symptoms (BDI‐II) or suicidal ideation (BSS). Change in perfectionistic concerns (FMPS‐CM) predicted neither depressive symptoms (BDI‐II) nor suicidal ideation (BSS) at 18‐month follow‐up.

## 4 Discussion

The present study investigated the association of perfectionism, more specifically perfectionistic concerns and perfectionistic strivings, with treatment outcome in MCT for depression and suicidal ideation (D‐MCT/S). It extends previous research by examining whether perfectionism predicts treatment outcome in a metacognitive treatment for depression. Considering the small sample size of this study, which is based on secondary analyses of a previous study, results should be considered preliminary.

Perfectionistic concerns (FMPS‐CM) at baseline were significantly related to depression (BDI‐II), but not suicidal ideation (BSS), as treatment outcomes. Surprisingly, this was a negative association, meaning greater perfectionistic concerns at the beginning of therapy were associated with less severe depression symptoms across time. This is in stark contrast to previous studies showing an impeding effect of perfectionism in the treatment of different disorders (e.g., Kyrios et al. 2015; Mitchell et al. 2013; Welch et al. 2020), as well as consistent evidence for cross‐sectional associations between perfectionistic concerns and psychopathology overall (for meta‐analyses, see Callaghan et al. 2023; Limburg et al. 2017; Lunn et al. 2023; Stackpole et al. 2023). More specifically in regard to depression, previous studies show an association between greater baseline perfectionism and poorer treatment outcome for depression (Blatt et al. 1995, 1998; Hawley et al. 2006, 2022; Hewitt et al. 2020; Jacobs et al. 2009; Marshall et al. 2008; Zuroff et al. 2000) and suicidal ideation (Beevers and Miller 2004; Jacobs et al. 2009). Similarly surprising was the finding that reductions in perfectionistic concerns (FMPS‐CM) during treatment did not significantly predict reductions in depressive symptoms (BDI‐II) or suicidal ideation (BSS) at 4‐week follow‐up. This is in contrast to a previous study showing an effect of reduced perfectionism on depression outcome (Hawley et al. 2006), which implicated the reduction of perfectionism as a promising process of therapeutic change. Several reasons could account for the discrepant findings.

First, it is important to consider methodological limitations. In light of differing results between preregistered and sensitivity analyses, we are hesitant to draw strong conclusions from current results. On the one hand, our sample size was relatively small compared to previous studies, so that a limited power may have led to nonsignificant results. On the other hand, the choice of symptom measures may have impacted our results. In contrast to the analyses presented here, our preregistered analyses (for details, see Supporting Information) revealed that the specific nature of the association between perfectionistic concerns (FMPS‐CM) at the beginning of treatment and depressive symptoms (BDI‐II) over time appears to partially depend on which measures are used to control for earlier symptom severity. Whereas preregistered analyses used a different depression measure (HDRS‐17) to control for earlier symptom severity, current analyses used a control measure identical to the outcome measure (BDI‐II). The preregistered approach, using the HDRS‐17 as the control, changed results insofar as that baseline perfectionistic concerns (FMPS‐CM) no longer significantly predicted depressive symptoms (BDI‐II) across time, whereas change in perfectionistic concerns (FMPS‐CM) now significantly predicted depressive symptoms (BDI‐II) at follow‐up. One explanation may be found in the specific items of the respective control measures: the BDI‐II includes items on self‐worth (“feeling like a loser,” “feeling disappointed in yourself,” “criticizing yourself,” “feeling worthless”) which the HDRS‐17 does not. Thus, a stronger overlap between the BDI‐II and the FMPS‐CM compared to the HDRS‐17 and the FMPS‐CM is possible. In addition, measure modality may have an impact, seeing as the BDI‐II is delivered as a self‐report questionnaire, whereas the HDRS‐17 is an interview administered by a clinician. These discrepancies between current and preregistered analyses show that perfectionistic concerns could play a role in depression treatment outcome, but that further investigation is needed to determine whether baseline perfectionism or change in perfectionism during treatment will prove to be more relevant, or whether future studies should take a combination of both into account.

Second, exploratory analyses emphasized the importance of using a measure designed to consider the multidimensionality of perfectionism. When adding perfectionistic strivings (FMPS‐PS) as a predictor on top of perfectionistic concerns (FMPS‐CM), perfectionistic concerns (FMPS‐CM) was no longer a significant negative predictor of depressive symptoms (BDI‐II) in current analyses (using the BDI‐II as the control measure). It could be argued that controlling perfectionistic concerns for perfectionistic strivings leaves only the self‐criticism component, without the achievement‐striving component. Whereas the former consistently appears to be positively associated with symptoms across several different disorders (Callaghan et al. 2023; Limburg et al. 2017; Lunn et al. 2023; Stackpole et al. 2023), the latter has yielded associations with both positive and negative outcomes (Stoeber and Gaudreau 2017). It is as of yet unclear whether perfectionistic strivings could act as a non‐harmful or even helpful component of perfectionism in psychotherapy. In the treatment of anorexia nervosa, for instance, one study found that greater perfectionistic strivings predicted faster weight gain (De Cuyper et al. 2019). Without separating the two components, both aspects of perfectionism may have been conflated in our models, which might explain why perfectionistic concerns (FMPS‐CM) appeared to have a beneficial effect in current results. Instead, future research should follow recent calls for differentiating between and statistically separating the effects of perfectionistic strivings and perfectionistic concerns (Stoeber and Gaudreau 2017). For this purpose, the FMPS appears better suited than unidimensional measures as used by the vast majority of previous studies.

Third, aside from methodological considerations, a cautious interpretation of current findings may be found in relation to the type of treatment provided. Previous findings showing a detrimental effect of perfectionism on treatment outcome were based on patients receiving “classic” CBT for depression. In the current study on patients receiving MCT, no such detrimental effects were found in either main or preregistered analyses. Whereas D‐MCT/S draws on CBT techniques, it additionally encourages patients to adopt a more distanced perspective when observing one's own thoughts and feelings, including perfectionistic beliefs. D‐MCT/S in particular includes a module which directly challenges perfectionistic beliefs. It is possible that such an approach may encourage patients to be more open towards exercises regardless of their outcome. A similar finding from research into treatments of obsessive‐compulsive disorder (OCD) would support this interpretation: Despite evidence for the impeding effect of perfectionism in CBT treatments for OCD (e.g., Kyrios et al. 2015), this effect was not found when investigating MCT and mindfulness‐based cognitive therapy for OCD (Claus et al. 2023). This is highly speculative, however, seeing as evidence on the role of perfectionism in newer treatment approaches such as MCT is scarce. Considering that patients in this current sample received not only D‐MCT/S (two sessions per week), but a comprehensive inpatient program including medication, it is unlikely that current results would be specific to D‐MCT/S as such. Additionally, it must be noted that on average, neither perfectionism dimension could be significantly reduced from baseline to posttreatment in this current sample, possibly due to the small number of participants for whom perfectionism measures were available at posttreatment (*n* = 29). Larger samples may reveal significant reductions of perfectionism and possibly larger effects of change in perfectionism on symptom severity in return. To draw conclusions on whether MCT and similar treatments could be better suited than traditional CBT for highly perfectionistic patients, future studies must provide a direct and preferably randomized comparison of CBT and MCT.

Lastly, none of the reported results yielded significant associations between perfectionism and suicidal ideation. This contrasts previous findings on the impeding role of baseline perfectionism in the treatment of suicidal ideation (Beevers and Miller 2004; Jacobs et al. 2009), as well as the association between perfectionism and suicidal ideation in general (see Smith et al. 2018 for a meta‐analysis). Seeing as previously reported effects tend to be small to moderate, our sample size might have been too small to detect such effects. In addition, it should be noted that suicidal ideation has proven more stable over time (Joiner 2002; Joiner et al. 2001) than depressive symptoms. This would mean less variance in the data and thus a smaller chance of finding prediction effects than when using depressive symptoms as the outcome instead. Another explanation for these results could be that we investigated D‐MCT/S as an intervention which openly addresses suicidal ideation in two dedicated group sessions. It is possible that this aspect of the treatment might reduce suicidal ideation more directly and effectively than the reduction of perfectionism, although this was not tested with the current data.

### 4.1 Strengths & Limitations

Results of the current study offer new insights into the role of perfectionism in the treatment of depression and suicidal ideation, extending findings beyond traditional CBT treatments. Analyses used data from a clinical sample with confirmed depression diagnosis and included the investigation of suicidal ideation as a common symptom of depression. We employed a specific perfectionism measure to represent multidimensionality of the concept and differentiate between perfectionistic concerns and perfectionistic strivings. The study was preregistered before data analysis.

However, some limitations need to be considered when interpreting these results. First, and most importantly, results must be considered preliminary as they are based on secondary analyses on a relatively small sample. Since we used pre‐existing data, no a priori power analysis was conducted. Seeing as “observed power” calculations are known to yield misleading results (Zhang et al. 2019), we also decided against a post‐hoc analysis. This means we cannot judge the statistical power of the analyses presented here. Power issues might have particularly impacted results for the effect of change in perfectionism on follow‐up outcomes, as the required data was available for a smaller subsample only (not all participants who took part in the posttreatment assessment were administered the FMPS). Secondly, the choice of measures for outcomes and controls clearly impacted our results, as seen in the difference between current and preregistered results. The impact of this particular decision in the study planning process should be considered carefully and tested further in future studies. Thirdly, treatment was provided in a largely controlled inpatient setting, as part of a treatment program including medication. We cannot strictly speak for the specificity of our results for D‐MCT/S, as a group treatment delivered bi‐weekly among several different treatment options, nor can results be generalized to outpatient treatment of depression and suicidal ideation. Lastly, we used data only from patients who took part in a minimum of four sessions, to ensure that they had received at least half of the intended treatment. However, it is possible that perfectionism could also impact drop‐out, thus impeding treatment outcome in a way we could not measure in this current study. Whereas we found no significant effect of perfectionism on number of sessions attended in this sample, this may have been due to limited power. Future studies in larger samples should consider taking drop‐out into account.

## 5 Conclusions

Taken together, these preliminary findings highlight the need for further investigation into the role of perfectionism in treatment effects in depression and suicidal ideation. Whereas sensitivity analyses revealed a certain reliance of effects on choice of control measures, results overall indicated that perfectionism appears to play a role in treatment outcomes, which must be investigated in greater detail in future studies. Results suggest that pretreatment levels of perfectionism may not have a strong obstructive effect on treatment outcomes in metacognitive treatments such as D‐MCT/S. However, the contrast to previous findings may also be due to different measures used or indeed limited power. In this context, the differentiation between perfectionistic concerns and perfectionistic strivings will require particular attention.

## Author Contributions

**Nathalie Claus:** methodology, formal analysis, writing – original draft. **Jakob Scheunemann:** investigation, writing – review and editing. **Sönke Arlt:** investigation, writing – review and editing. **Judith Peth:** investigation, writing – review and editing. **Jürgen Gallinat:** investigation, writing – review and editing. **Barbara Cludius:** writing – review and editing, supervision. **Lena Jelinek:** conceptualization, methodology, writing – review and editing, funding acquisition, resources, supervision.

## Ethics Statement

All procedures contributing to this study comply with the ethical standards of the relevant national and institutional committees on human experimentation and with the 1964 Helsinki Declaration and its later amendments.

## Consent

Informed consent was obtained from all participants included in the study.

## Conflicts of Interest

The authors declare no conflicts of interest.

## Supporting information


**Supporting Information Table S1:** Correlation Matrix. **Table S2:** Results of the final multi‐level models for preregistered analyses. **Table S3:** Exploratory analyses using both FMPS‐PS and FMPS‐CM as predictors. **Table S4:** Exploratory analyses using outcomes at 18‐month follow‐up.

## Data Availability

The data that support the findings of this study have been anonymized and made openly available on OSF at https://osf.io/fksur/ [https://doi.org/10.17605/OSF.IO/FKSUR]. This is also where data analysis was preregistered.
