# Supplementary material for: Perfectionism as Possible Predictor for Treatment Success: Preliminary Data From Metacognitive Training for Depression and Suicidal Ideation in an Inpatient Sample
Source: J Clin Psychol. 2025 Aug 7;81(11):1166–77. doi: 10.1002/jclp.70027 (PMC12501822; doi:10.1002/jclp.70027)
Supplement: Supplementary file 1 — Supporting Information Table S1: Correlation Matrix. Table S2: Results of the final multi‐level models for preregistered analyses. Table S3: Exploratory analyses using both FMPS‐PS and FMPS‐CM as predictors. Table S4: Exploratory analyses using outcomes at 18‐month follow‐up. [file JCLP-81-1166-s001.docx]

**Supporting Information**

**1. Imputation of missing values**

Where calculation of questionnaire sum scores was required, the proportion of NAs per questionnaire was checked, similar to Wilhelm and colleagues (2015). If more than 10% of item responses within a given questionnaire from one participant were missing, data on this particular questionnaire from this participant were excluded from analysis. If fewer than 10% of item responses were missing, Little’s test (Little, 1988) was used to determine whether item responses were missing at random. If so, NAs were imputed using person mean imputation. If Little’s test revealed item responses were missing not at random, no values were imputed and instead sum scores were calculated using all responses given.

**2. Assumed model equations**

***2.1 Hypotheses 1 and 3: multi-level model***

Symptom*_ij_* ~ *b*_0_*_i_* + *b*_1_*_i_* * PerfectionismBaseline*_i_* + *b*_2_*_i_* * SymptomBaseline*_i_* + *b*_3_*_i_* * Time*_ij_* + *b*_4_ * (Perfectionism x Time)*_ij_* + *ɛ_ij_*

*i*: participants, *j*: time points; with *b_0i_* = *b*_0_ + *u*_0_*_i_*, *b*_1_*_i_* = *b*_1_ *+ u*_1_*_i_*, *b*_2_*_i_* = *b*_2_ *+ u*_2_*_i_*, *b*_3_*_i_* = *b*_3_ *+ u*_3_*_i_*

Symptom*_ij_*/SymptomBaseline*_i_*: depression (1) or suicidality (3)

PerfectionismBaseline*_i_*: perfectionistic concerns

***2.2 Hypotheses 2 and 4: multi-level model***

SymptomFU*_i_* ~ *b*_0_*_i_* + *b*_1_*_i_* * PerfChange*_i_* + *b*_2_*_i_* * SymptomPost*_i_* + *ɛ_i_*

*i*: participants; with *b_0i_* = *b*_0_ + *u*_0_*_i_*, *b*_1_*_i_* = *b*_1_ *+ u*_1_*_i_*, *b*_2_*_i_* = *b*_2_ *+ u*_2_*_i_*

SymptomFU*_i_*/SymptomPost*_i_*: depression (2) or suicidality (4)

PerfChange*_i_*: residuals of perfectionistic concerns

**3. Bivariate correlations between variables**

Table S1: Correlation Matrix

| Variable | 1. | 2. | 3. | 4. | 5. | 6. | 7. | 8. | 9. | 10. | 11. | | 12. | | 13. | | 14. | | 15. | | 16. | |
| --- | --- | --- | --- | --- | --- | --- | --- | --- | --- | --- | --- | --- | --- | --- | --- | --- | --- | --- | --- | --- | --- | --- |
| 1. FMPS-CM pre | 1 |  |  |  |  |  |  |  |  |  | |  | |  | |  | |  | |  | |  |
| 2. FMPS-PS pre | .63^***^ | 1 |  |  |  |  |  |  |  |  | |  | |  | |  | |  | |  | |  |
| 3. FMPS-CM post | .76^***^ | .69^***^ | 1 |  |  |  |  |  |  |  | |  | |  | |  | |  | |  | |  |
| 4. FMPS-PS post | .51^**^ | .86^***^ | .72^***^ | 1 |  |  |  |  |  |  | |  | |  | |  | |  | |  | |  |
| 5. HDRS-17 pre | .17 | .11 | .15 | .05 | 1 |  |  |  |  |  | |  | |  | |  | |  | |  | |  |
| 6. HDRS-17 post | .12 | .20 | .38^*^ | .25 | .45^**^ | 1 |  |  |  |  | |  | |  | |  | |  | |  | |  |
| 7. HDRS-17 FU1 | .02 | .21 | .36 | .27 | .43^**^ | .74^***^ | 1 |  |  |  | |  | |  | |  | |  | |  | |  |
| 8. HDRS-17 FU2 | .29 | .25 | .28 | .30 | .45^**^ | .58^***^ | .60^***^ | 1 |  |  | |  | |  | |  | |  | |  | |  |
| 9. BDI-II pre | .42^**^ | .16 | .43^*^ | -.01 | .63^***^ | .52^***^ | .35^*^ | .39^*^ | 1 |  | |  | |  | |  | |  | |  | |  |
| 10. BDI-II post | .03 | -.06 | .45^*^ | .13 | .43^**^ | .64^***^ | .51^***^ | .31 | .73^***^ | 1 | |  | |  | |  | |  | |  | |  |
| 11. BDI-II FU1 | .05 | .04 | .51^**^ | .21 | .42^**^ | .66^***^ | .70^***^ | .50^**^ | .59^***^ | .79^***^ | | 1 | |  | |  | |  | |  | |  |
| 12. BDI-II FU2 | .24 | .23 | .31 | .34 | .48^**^ | .54^***^ | .61^***^ | .86^***^ | .49^**^ | .30 | | .50^**^ | | 1 | |  | |  | |  | |  |
| 13. BSS pre | .15 | .05 | .06 | .14 | .41^**^ | .40^**^ | .38^**^ | .47^**^ | .47^***^ | .38^**^ | | .39^**^ | | .49^**^ | | 1 | |  | |  | |  |
| 14. BSS post | -.03 | .04 | .08 | .26 | .23 | .49^***^ | .39^**^ | .35^*^ | .36^*^ | .51^***^ | | .46^**^ | | .37^*^ | | .80^***^ | | 1 | |  | |  |
| 15. BSS FU1 | .11 | .09 | .27 | .28 | .31^*^ | .60^***^ | .52^***^ | .57^***^ | .53^***^ | .62^***^ | | .70^***^ | | .60^***^ | | .78^***^ | | .80^***^ | | 1 | |  |
| 16. BSS FU2 | .08 | .16 | .03 | .29 | .18 | .44^**^ | .50^**^ | .64^***^ | .22 | .20 | | .34^*^ | | .71^***^ | | .73^***^ | | .72^***^ | | .76^***^ | | 1 |

*Note.* Time points: pre = baseline measurement; post = post-treatment measurement; FU1/FU2 = follow-ups at 4 weeks, 18 months after end of treatment. FMPS-CM = Frost Multidimensional Perfectionism Scale, subscale “concern over mistakes”. FMPS-PS = Frost Multidimensional Perfectionism Scale, subscale “personal standards”. HDRS-17 = Hamilton Depression Rating Scale. BDI-II = Beck Depression Inventory-II. BSS = Beck Scale for Suicide Ideation. Asterisks signify level of significance: * for p < .05, ** for p < .01, *** for p < .001.

Multicollinearity between predictors was examined for each of the final models, using the variance inflation factor (VIF). For all predictors in all models, VIF ≤ 2, suggesting small to moderate correlation between predictor variables.

**4. Statistical values used for model selection**

***4.1 Hypothesis 1***

In the basic model, patients explained a large proportion of the variance in outcome, ICC = 0.60. When contrasting models, adding the predictors perfectionism at baseline, symptoms at baseline and time improved model fit compared to the baseline model (log likelihood value = 23.39, *p* < .0001). Including the interaction term (perfectionism and time) did not improve model fit when compared to a model without the interaction (log likelihood value = 0.96, *p* = .33). Further, the model with a random slope for time fit the data better than a model without random slopes (log likelihood value = 11.66, *p* = .003), whereas models with random slopes for baseline perfectionism or baseline symptoms showed no significant improvement of model fit (log likelihood value = 2.24, *p* = .33). Finally, the model with the best fit included the predictors concern over mistakes (FMPS-CM), symptoms (BDI-II) at baseline, and time, but not the interaction between concern over mistakes and time. Further, the model with a random slope for time but not for baseline concern over mistakes and baseline symptoms fit the data best.

***4.2 Hypothesis 2***

In the basic model, patients explained a large proportion of the variance in outcome, ICC = 0.88. When contrasting models, adding the two predictors improved model fit compared to the baseline model (log likelihood value = 14.57, *p* = .0001). Adding random slopes was not possible as there were fewer observations than random effects. Finally, the model with the best fit included the predictors change in concern over mistakes from pre- to post-treatment (FMPS-CM) and depressive symptom severity at post-treatment (BDI-II), with no random slopes.

***4.3 Hypothesis 3***

When contrasting models using the BSS as outcome, patients explained a large proportion of the variance in outcome in the basic model, ICC = 0.78. Adding the predictors perfectionism at baseline, symptoms at baseline and time improved model fit compared to the baseline model (log likelihood value = 6.18, *p* = .01). Including the interaction between perfectionism and time did not improve model fit when compared to a model without the interaction (log likelihood value = 0.01, *p* = .92). Similarly, adding random slopes for any of the predictor variables did not improve fit when compared to a model without random slopes (log likelihood value = 0.32, *p* = .85; log likelihood value = 1.12, *p* = .57; log likelihood value = 0.32, *p* = .85). Finally, the model with the best fit included the predictors concern over mistakes (FMPS-CM) at baseline, suicidal ideation (BSS) at baseline, and time, but not the interaction between concern over mistakes and time.

***4.4 Hypothesis 4***

When contrasting models using the BSS as outcome, patients explained a large proportion of the variance in outcome in the basic model, ICC = 0.88. Adding the two predictors improved model fit compared to the baseline model (log likelihood value = 14.55, *p* = .0001). Adding random slopes was not possible as there were fewer observations than random effects. Finally, the model with the best fit included the predictors pre-post change in concern over mistakes (FMPS-CM) and suicidal ideation (BSS) at post-treatment, with no random slopes.

**5. Equations for final multi-level models**

***5.1 Hypothesis 1 (outcome BDI-II)***

Symptom*_ij_* ~ *b*_0_*_i_* + *b*_1_ * PerfectionismBaseline*_i_* + *b*_2_ * SymptomBaseline*_i_* + *b*_3_*_i_* * Time*_ij_* + *ɛ_ij_*

*i*: participants, *j*: time points; with *b_0i_* = *b*_0_ + *u*_0_*_i_*, *b*_3_*_i_* = *b*_3_ *+ u*_3_*_i_*

***5.2 Hypothesis 3 (outcome BSS)***

Symptom*_ij_* ~ *b*_0_*_i_* + *b*_1_ * PerfectionismBaseline*_i_* + *b*_2_ * SymptomBaseline*_i_* + *b*_3_ * Time*_ij_* + *ɛ_ij_*

*i*: participants, *j*: time points; with *b_0i_* = *b*_0_ + *u*_0_*_i_*

***5.3 Hypotheses 2 and 4 (outcomes BDI-II and BSS)***

SymptomFU*_i_* ~ *b*_0_*_i_* + *b*_1_ * PerfChange*_i_* + *b*_2_ * SymptomPost*_i_* + *ɛ_i_*

*i*: participants; with *b_0i_* = *b*_0_ + *u*_0_*_i_*

**6. Skewness and kurtosis**

***6.1 FMPS-CM***

Jarque test (from the *moments* package) revealed no significant deviations from normal distribution (*p* = .21), with a minor left-skew (-0.21) and kurtosis < 3 (1.84).

***6.2 FMPS-PS***

Jarque test revealed no significant deviations from normal distribution (*p* = .13), with a minor left-skew (-0.66) and kurtosis < 3 (2.51).

***6.3 BDI-II***

Jarque test revealed no significant deviations from normal distribution (*p* = .61), with a minor right-skew (0.30) and kurtosis < 3 (2.66).

***6.4 HDRS-17***

Jarque test revealed no significant deviations from normal distribution (*p* = .99), with a minor right-skew (0.04) and kurtosis < 3 (2.97).

***6.5 BSS***

Jarque test revealed no significant deviations from normal distribution (*p* = .10), with a minor right-skew (0.37) and kurtosis < 3 (1.70).

**7. Results of preregistered analyses**

Table S2: Results of the final multi-level models for preregistered analyses

|  | *n* | β^a^ | *95% CI* | *SE* | *t* | *p* |
| --- | --- | --- | --- | --- | --- | --- |
| **H1: Dependent variable: depressive symptom severity (BDI-II) across time** | 49 |  |  |  |  |  |
| Intercept |  | 33.61 | 30.98 – 36.24 | 1.34 | 25.03 | **<.001** |
| Perfectionistic concerns at baseline (FMPS-CM) |  | 0.30 | 0.01 – 0.60 | 0.15 | 2.06 | .045 |
| Depressive symptoms at baseline (HDRS-17) |  | 1.03 | 0.62 – 1.43 | 0.20 | 5.06 | **<.001** |
| Time |  | -1.03 | -1.48 – -0.58 | 0.23 | -4.48 | **<.001** |
| **H2: Dependent variable: depressive symptom severity (BDI-II) at four-week follow-up** | 29 |  |  |  |  |  |
| Intercept |  | 35.12 | 30.68 – 39.57 | 2.28 | 15.38 | **<.001** |
| Change in perfectionistic concerns (FMPS-CM) |  | 1.00 | 0.29 – 1.71 | 0.36 | 2.75 | **0.011** |
| Depressive symptoms at post (HDRS-17) |  | 1.23 | 0.65 – 1.81 | 0.30 | 4.12 | **<.001** |
| **H3: Dependent variable: severity of suicidal ideation (BSS) across time** | 49 |  |  |  |  |  |
| Intercept |  | 10.70 | 8.08 – 13.32 | 1.34 | 7.99 | **<.001** |
| Perfectionistic concerns at baseline (FMPS-CM) |  | -0.14 | -0.44 – 0.16 | 0.15 | -0.93 | 0.355 |
| Depressive symptoms at baseline (BDI-II) |  | 0.44 | 0.22 – 0.66 | 0.11 | 3.90 | **<.001** |
| Time |  | -0.32 | -0.58 – -0.06 | 0.13 | -2.41 | **<.018** |
| **H4: Dependent variable: severity of suicidal ideation (BSS) at four-week follow-up** | 28 |  |  |  |  |  |
| Intercept |  | 13.96 | 10.51 – 17.40 | 1.77 | 7.88 | **<.001** |
| Change in perfectionistic concerns (FMPS-CM) |  | -0.09 | -0.72 – 0.54 | 0.32 | -0.27 | 0.787 |
| Depressive symptoms at post (BDI-II) |  | 0.57 | 0.30 – 0.84 | 0.14 | 4.15 | **<.001** |

*Note.* FMPS_CM = Frost Multidimensional Perfectionism Scale, “concern over mistakes” subscale. FMPS_PS = Frost Multidimensional Perfectionism Scale, “personal standards” subscale. HDRS-17 = Hamilton Depression Rating Scale-17. BSS = Beck Suicide Ideation Scale. BDI-II = Beck Depression Inventory II. ^a^ β (= fixed effect) denotes magnitude of change in the outcome variable as the predictor increases by one point relative to grand-mean at baseline. Bold *p* values denote significance below α = 0.025 (Bonferroni-corrected for multiple comparisons).

**8. Results of exploratory analyses**

### *8.1 Effect of Baseline Perfectionism (FMPS-CM & FMPS-PS) on Symptom Severity (Exploratory 1 and 2)*

We introduced the perfectionism dimension personal standards (FMPS-PS) at baseline as an additional predictor to the final models as described above (H1 and H3).

Table S3: Exploratory analyses using both FMPS-PS and FMPS-CM as predictors

|  | *n* | β^a^ | *95% CI* | *SE* | *t* | *p* |
| --- | --- | --- | --- | --- | --- | --- |
| **Exploratory 1: Dependent variable: depressive symptom severity (BDI-II) across time** | 49 |  |  |  |  |  |
| Intercept |  | 32.27 | 31.36 – 33.18 | 0.47 | 69.10 | **<.001** |
| Perfectionistic concerns at baseline (FMPS-CM) |  | -0.14 | -0.29 – 0.00 | 0.07 | -1.92 | 0.061 |
| Perfectionistic strivings at baseline (FMPS-PS) |  | 0.01 | -0.19 – 0.21 | 0.10 | 0.09 | 0.930 |
| Depressive symptoms at baseline (BDI-II) |  | 1.01 | 0.93 – 1.10 | 0.04 | 23.75 | **<.001** |
| Time |  | -1.05 | -1.49 – -0.61 | 0.23 | -4.63 | **<.001** |
| **Exploratory 2: Dependent variable: severity of suicidal ideation (BSS) across time** | 49 |  |  |  |  |  |
| Intercept |  | 10.73 | 9.28 – 12.18 | 0.74 | 14.45 | **<.001** |
| Perfectionistic concerns at baseline (FMPS-CM) |  | -0.07 | -0.23 – 0.08 | 0.77 | -0.97 | 0.339 |
| Perfectionistic strivings at baseline (FMPS-PS) |  | 0.08 | -0.15 – 0.30 | 0.11 | 0.67 | 0.509 |
| Suicidal ideation at baseline (BSS) |  | 0.87 | 0.77 – 0.97 | 0.05 | 17.48 | **<.001** |
| Time |  | -0.32 | -0.58 – -0.05 | 0.13 | -2.34 | **<.05** |

*Note.* FMPS_CM = Frost Multidimensional Perfectionism Scale, “concern over mistakes” subscale. FMPS_PS = Frost Multidimensional Perfectionism Scale, “personal standards” subscale. BSS = Beck Suicide Ideation Scale. BDI-II = Beck Depression Inventory II. ^a^ β (= fixed effect) denotes magnitude of change in the outcome variable as the predictor increases by one point relative to grand-mean at baseline. Bold p values denote significance below α = 0.05.

Using depressive symptoms (BDI-II) as outcome, neither personal standards (FMPS-PS) nor concern over mistakes (FMPS-CM) at baseline proved to be a significant predictor. However, depressive symptoms (BDI-II) at baseline and time showed significant effects on depressive symptoms (BDI-II) across time points; that is, higher depressive symptoms (BDI-II) at baseline were associated with higher depressive symptoms (BDI-II), and with every week since baseline, depressive symptoms (BDI-II) decreased. Fixed effects explained 65.6% of variance, with the entire model (including random effects) explaining 91.4% (σ2=19.13, τ00=0.61, τ11=1.87, ρ01=.95, ICC=0.75). This model used data from all 49 participants.

Using suicidality (BSS) as outcome, neither personal standards (FMPS-PS) nor concern over mistakes (FMPS-CM) at baseline proved to be a significant predictor. Only suicidal ideation (BSS) at baseline and time showed significant effects on suicidality (BSS) across time points; that is, higher suicidal ideation (BSS) at baseline was associated with more severe suicidality (BSS), and with every week since baseline, suicidality (BSS) decreased. Fixed effects explained 75.7% of variance (σ2=28.06, τ00=0.01, ICC=0.01). This model used data from all 49 participants.

### *8.2* *Effect of Change in Perfectionism (FMPS-CM) on Depressive Symptom Severity and Suicidality (Exploratory 3 and 4)*

We examined the effect of pre-post change in perfectionism (FMPS-CM) on both symptom outcomes (BDI-II and BSS, respectively) using data from an 18-month follow-up instead of the four-week follow-up as described above (H2 and 4).

Table S4: Exploratory analyses using outcomes at 18-month follow-up

|  | *n* | β^a^ | *95% CI* | *SE* | *t* | *p* |
| --- | --- | --- | --- | --- | --- | --- |
| **Exploratory 3: Dependent variable: depressive symptom severity (BDI-II) at 18-month follow-up** | 22 |  |  |  |  |  |
| Intercept |  | 32.12 | 25.05 – 39.19 | 3.64 | 8.84 | **<.001** |
| Change in perfectionistic concerns (FMPS-CM) |  | -0.09 | -1.46 – 1.27 | 0.70 | -0.13 | 0.895 |
| Depressive symptoms at post (HDRS-17) |  | 1.30 | 0.42 – 2.19 | 0.46 | 2.86 | **0.010** |
| **Exploratory 4: Dependent variable: severity of suicidal ideation (BSS) at 18-month follow-up** | 22 |  |  |  |  |  |
| Intercept |  | 10.61 | 5.35 – 15.87 | 2.70 | 3.92 | **.001** |
| Change in perfectionistic concerns (FMPS-CM) |  | -0.46 | -1.63 – 0.71 | 0.60 | -0.77 | 0.451 |
| Depressive symptoms at post (BDI-II) |  | 0.22 | -0.17 – 0.61 | 0.20 | 1.10 | 0.284 |

*Note.* FMPS_CM = Frost Multidimensional Perfectionism Scale, “concern over mistakes” subscale. HDRS-17 = Hamilton Depression Rating Scale-17. BSS = Beck Suicide Ideation Scale. BDI-II = Beck Depression Inventory II. ^a^ β (= fixed effect) denotes magnitude of change in the outcome variable as the predictor increases by one point relative to grand-mean at baseline. Bold p values denote significance below α = 0.05.

Using depressive symptoms (BDI-II) as outcome, patients explained a large proportion of the variance in outcome in the basic model, ICC = 0.88. After contrasting models, the model with the best fit included the predictors change in concern over mistakes from pre- to post-treatment (FMPS-CM) and depressive symptom severity at post-treatment (HDRS-17), with no random slopes. The final model showed that pre-post change in concern over mistakes (FMPS-CM) had no significant effect, with only depressive symptom severity (HDRS-17) at post-treatment predicting depressive symptoms (BDI-II) at 18-month follow-up; that is, higher depression scores (HDRS-17) at post-treatment predicted higher depression scores (BDI-II) at 18-month follow-up. Fixed effects explained 81% of variance (σ2=23.05, τ00=163.90, ICC=0.88). This model used data from 22 participants (complete FMPS data at baseline and post-treatment as well as BDI-II data at 18-months follow-up).

Using suicidality (BSS) as outcome, patients explained a large proportion of the variance in outcome in the basic model, ICC = 0.88. After contrasting models, the baseline model with fixed intercept only yielded the best fit. However, for exploratory analysis, we inspected model parameters for the model including the predictors pre-post change in concern over mistakes (FMPS-CM) and depressive symptom severity (BDI-II) at post-treatment, with no random slopes. This model showed neither pre-post change in concern over mistakes (FMPS-CM) nor depressive symptoms (BDI-II) at post-treatment had significant effects on suicidality (BSS) at 18-month follow-up. Fixed effects explained 36% of variance (σ2=14.91, τ00=106.05, ICC=0.88). This model used data from 22 participants (complete FMPS data at baseline and post-treatment as well as BSS data at 18-month follow-up).
